# Ecological Grief as a Response to Environmental Change: A Mental Health Risk or Functional Response?

**DOI:** 10.3390/ijerph18020734

**Published:** 2021-01-16

**Authors:** Hannah Comtesse, Verena Ertl, Sophie M. C. Hengst, Rita Rosner, Geert E. Smid

**Affiliations:** 1 Section of Clinical and Biological Psychology, Catholic University Eichstaett-Ingolstadt, 85072 Eichstaett, Germany; hannah.comtesse@ku.de (H.C.); Verena.Ertl@ku.de (V.E.); rita.rosner@ku.de (R.R.); 2 ARQ National Psychotrauma Centre, 1112 XE Diemen, The Netherlands; S.Hengst@centrum45.nl; 3 Department of Humanist Chaplaincy Studies, University of Humanistic Studies, 3512 HD Utrecht, The Netherlands

**Keywords:** environmental change, climate change threats, ecological grief, environmental grief, climate grief, solastalgia, eco-anxiety, mental health, risk factor, adaptation

## Abstract

The perception of the impact of climate change on the environment is becoming a lived experience for more and more people. Several new terms for climate change-induced distress have been introduced to describe the long-term emotional consequences of anticipated or actual environmental changes, with ecological grief as a prime example. The mourning of the loss of ecosystems, landscapes, species and ways of life is likely to become a more frequent experience around the world. However, there is a lack of conceptual clarity and systematic research efforts with regard to such ecological grief. This perspective article introduces the concept of ecological grief and contextualizes it within the field of bereavement. We provide a case description of a mountaineer in Central Europe dealing with ecological grief. We introduce ways by which ecological grief may pose a mental health risk and/or motivate environmental behavior and delineate aspects by which it can be differentiated from related concepts of solastalgia and eco-anxiety. In conclusion, we offer a systematic agenda for future research that is embedded in the context of disaster mental health and bereavement research.

## 1. Introduction

Climate change is increasingly recognized to pose a variety of threats to human health, including mental health and well-being [1,2,3]. The mental health effects of climate change-related environmental changes may occur directly due to natural disasters and extreme weather events (e.g., as traumatic stress reactions due to floods or wildfires) or indirectly through long-term, mostly secondary stressors that foster mental disorders (e.g., less secure food supply, problematic process of reconstruction of housing or infrastructure, forced migration due to increasing temperatures). Moreover, the impacts of climate change on mental health range from acute (e.g., distress during or after floods or heatwaves) to chronic (e.g., distress due to permanent landscape changes after tornados or mountains’ loss of snow cover). 

The perception of the long-term impact of climate change on the environment (e.g., gradually changing weather patterns, deglaciation, deforestation) is a lived experience for more and more people. In addition to the acute and chronic impacts of direct exposure to natural disasters on mental health (e.g., stress reactions or PTSD due to wildfires), many people suffer the emotional consequences associated with anticipated or actual environmental changes by reacting with powerlessness, helplessness, despair, grief or uncertainty [2,4]. In a representative survey of the Greenland population, 15% of participants reported strong fear with regard to the changing climate, while hopelessness was reported by 6%, sadness and guilt by 7% and anger by 5% [5]. Other surveys showed that 56% of rural Australians were worried about climate change [6], while 29% of Americans were “very worried” about global warming and the majority of Americans were at least a “little worried” about extreme events in their local area such as extreme heat (64%) or droughts (60%) [7]. Meanwhile, even many European countries are gradually impacted by changes in weather patterns, species diversity and landscapes. A survey of perceptions of climate change in four European countries (France, Germany, Great Britain, Norway; [8]) found that about 30% of participants were “very or extremely worried” about climate change and that emotions of fear, outrage and guilt in relation to climate change were present in 8–42%. 

Over the past 15 years, several new terms for climate change-induced distress have been introduced to describe such long-term emotional impacts of climate change, e.g., [9,10,11], with ecological grief as a prime example. This has already been recognized in popular culture. For example, the BBC news published an article on “Climate grief: How we mourn a changing planet” in April 2020 [12] and the Guardian reported on “‘Solastalgia’: Arctic inhabitants overwhelmed by new form of climate grief” in October 2020 [13]. In research, however, there is a dearth of studies on ecological grief and its possible impacts on mental health. The purpose of this perspective article is to focus on the long-term impacts of climate change-related losses as the mourning of the loss of ecosystems, landscapes, species and ways of life is likely to become a more frequent experience around the world. Therefore, we introduce the concept of ecological grief and contextualize it within the field of bereavement. This article begins with an introduction of the ecological grief concept as it has been used to date, followed by a fictional case example of ecological grief in Central Europe. Then, we present the related concepts of solastalgia and eco-anxiety and suggest ways to differentiate the three concepts in order to gain more conceptual clarity. Then, we delineate the links of ecological grief to the bereavement literature and the state of research on ecological grief. We further provide a description of its possible effects on mental health and mental health risks as well as environmental behavior. Finally, we provide an agenda for future research that is embedded in the context of bereavement research and could improve the understanding of long-term climate change-related distress beyond the short- and long-term mental health consequences of direct exposure to disasters.

## 2. Conceptualization and Case Description of Ecological Grief

A loss represents a reduction in tangible or intangible resources, in which an emotional investment has been made in [14]. A systematic review on climate-related non-economic losses has shown that people are subject to intangible harm from climate change (e.g., disappearance of fauna and flora, loss of cropland and living spaces for animals, loss of ways of life, loss of personal identity constructed in relation to the physical environment; [15]). Ecological grief is the emotional response to such losses. Cunsolo and Ellis define it as “the grief felt in relation to experienced or anticipated ecological losses, including the loss of species, ecosystems and meaningful landscapes due to acute or chronic environmental change” [11] (p. 275). As such, ecological grief is a natural response to ecological loss, which is supposedly particularly pronounced in people who retain close relationships with the natural environment [11] such as foresters, farmers, mountaineers, divers or indigenous peoples, but may well be universal. 

Based on mainly qualitative data, three types of loss have been proposed to evoke ecological grief [11]. First, acute or past physical ecological loss means the disappearance, degradation or extinction of species, landscapes and ecosystems. For example, grief has been identified as a response to acute extreme weather events (e.g., hurricane; [16]) and gradual environmental changes (e.g., changing weather patterns; [17]). Second, loss of environmental knowledge refers to the disruption of personal and cultural identities that are constructed in relation to features and knowledge of the physical environment. Third, anticipated future loss relates to that of species, landscapes, ecosystems, ways of life or livelihoods.

Place attachment has been invoked as the theoretical foundation of ecological grief [11]. In this sense, the concept of place refers to a space that has acquired personal meaning and can be applied to aspects of the social and physical environment such as a house, neighborhood, landscape or natural environment (e.g., forests; [18]). Many people form an attachment to places, constructing part of their identity around it [18]. This could explain the disruption after ecological loss, resulting in a grief-like reaction as described in the fictional case of a mountaineer in Central Europe below.

### Fictional Case Description Of Ecological Grief

The 50-year-old A. is a mountaineer in the alpine region. His favorite mountain is affected by deglaciation and a loss of the snow cover. He describes that he had developed a relationship with the ice field, and in its diminishment, he feels the diminishment of himself. “I can barely look at the glacier, gleaming in sunshine. All I can see is melting; all I can see is what once was, what will not be.” He experiences the mountain without the glacier as naked and ugly. A. is still stunned by the lost landscape and longs for it. His thoughts circle around the loss and its consequences. He blames himself for non-climate friendly, past actions while he cannot accept the loss. He experiences sadness, bitterness, deep anxiety, guilt, and even shame about his lifestyle choices. He dreams about images of a deserted, uninhabitable world. He often looks at old pictures of him in the mountains to feel connected to the lost landscape, a feeling he describes as homesickness. “Whatever great feeling I had is gone.” He does not even know who he really is without the cherished environment and has a diminished sense of security and control. His thoughts are preoccupied with the wanton destruction of treasured landscapes around the globe, leaving only ravage and desolation. During periods of profound distress, he feels intense visceral pain and mental anguish. He has withdrawn from people he cares about and those with whom he used to spend his recreational time. He has the feeling that he holds no sense of purpose without his cherished natural environment. 

## 3. Ecological Grief and Related Concepts of Climate Change-Induced Distress 

Several new terms for climate change-induced distress have been introduced to describe long-term emotional consequences of environmental change e.g., [9,10,11], with ecological grief, solastalgia and eco-anxiety as key representatives. These terms refer to different emotional response patterns and timeframes of climate change effects (i.e., before, during and after actual environmental change). However, there is conceptual ambiguity with regard to the convergence and divergence of the concepts [11,19].

### 3.1. Solastalgia

The concept of solastalgia refers to a sense of desolation, detachment and grieving in response to losing an important place which is similar to homesickness whilst one is still at home [9,20]. It occurs when people are confronted with irrevocable changes to landscapes that they feel connected to. Place attachment features prominently in conceptual foundations of solastalgia [19]. Galway and colleagues defined solastalgia as “the distress caused by the unwelcome transformation of cherished landscapes resulting in cumulative mental, emotional and spiritual health impacts” [19] (p. 11). In this sense, solastalgia can be evoked by transformations of places (e.g., a specific location, region or territory) beyond the home environment, meaning a physical ecological loss of landscapes, even though it may have a gradual beginning due to slow-moving changes in the living environment [3]. Research on solastalgia is mostly based on qualitative interviews or the use of the solastalgia subscale of the Environmental Distress Scale (EDS) [21] in groups particularly exposed to changes in their home environment (e.g., survivors of natural disasters, individuals affected by rapid industrial development). The solastalgia subscale captures feelings of yearning, longing and sadness but also worrying with regard to impending environmental and lifestyle changes. Hence, the scale seems to assess grief with regard to actual ecological loss but also anxiety in response to anticipated, future loss.

### 3.2. Eco-Anxiety

Climate change-related environmental degradation and loss of species or ways of life can evoke emotional reactions even before its occurrence. Eco-anxiety (or climate change anxiety) is a response to impending threats by climate change (e.g., rising sea levels, desertification; [4,22,23]). The perception of the slow-moving impacts of climate change can evoke feelings of helplessness, hopelessness and uncertainty. Anxiety is an adaptive response to future-oriented, possible threats [24] that encompasses such aversive emotion states, cognitive worrying and physiological arousal and apprehension [25,26]. As such, eco-anxiety is an adaptive response to the threat of climate change which has been shown to be associated with pro-environmental behavioral engagement [4,27]. However, if eco-anxiety is characterized by severe and debilitating worrying, it can be maladaptive and possibly lead to the development of anxiety disorders [4,23]. Generalized anxiety disorder would be a prime candidate [4] as it involves uncontrollable worrying about possible future harm to loved ones and oneself. Research on eco-anxiety is mostly based on qualitative interviews or ad hoc items. The newly introduced questionnaire of climate change anxiety [4] could stimulate future research. The questionnaire captures cognitive worrying, physical symptoms such as problems concentrating, functional impairment as well as behavioral engagement. 

### 3.3. Differentiation of the Three Concepts

Solastalgia closely overlaps with ecological grief in terms of triggering conditions, phenomenology and conceptual foundations. Even though there has been a recent shift from the home environment to any cherished landscapes in conceptualizing solastalgia [19], it may be regarded as a sub-concept of ecological grief. Beyond landscapes, ecological grief could be evoked by all ecological losses (e.g., species, ecosystems, ways of life). Eco-anxiety is inherently future-oriented e.g., [4]. Therefore, anxiety can be expected to be the predominant reaction to anticipated future ecological loss as anticipated ecological loss refers in part to impending threats by climate change. We would regard ecological grief as a response to actual and past ecological loss or a reaction to future situations that trigger the current loss (e.g., not being able to teach one’s children skiing on the same mountain where oneself once learned how to ski) and thus expect few shared phenomenological features with eco-anxiety.

## 4. Ecological Grief and Bereavement Research

### 4.1. Grief in the Context of Bereavement Research

Grief is a universal and natural response to separation or loss and encompasses a range of emotional (e.g., yearning, bitterness), cognitive (e.g., preoccupation with the loss, diminished identity) and behavioral reactions (e.g., withdrawal from social and recreational activities). A feeling of yearning is paramount, which is focused on a desire for a place, thing or person that was highly treasured [28]. Yearning has been identified in job loss [29], romantic breakups [30], homesickness [31] and as a cardinal symptom of a pathological type of grief after the death of a loved person, prolonged grief disorder (PGD; [32]). Moreover, yearning and other emotional, cognitive and behavioral grief reactions feature prominently in the case of the mountaineer described under 2.1. Attachment to other persons is a core human goal and is fundamental to emotional reactions after separation [33]. Therefore, attachment features prominently as a scientific explanation for grief reactions and PGD [34,35,36]. Likewise, place attachment has been discussed as a scientific framework for homesickness [37] as also for ecological grief [11]. 

The concept of PGD has spurred research on the determinants, manifestations, underlying processes and consequences of grief following bereavement over the last two decades, e.g., [36,38,39]. The majority of bereaved persons experience a period of painful emotions accompanied by more or less debilitating psychological and physical symptoms after which they are able to recover [39]. Only one in ten bereaved persons develop pathological grief reactions that interfere with daily life for an extended period of time following the loss [40]. Research has shown that PGD can be discriminated from PTSD, depression and anxiety disorders e.g., [41,42]. In general, grief is a highly individualized reaction that depends not only on the characteristics of the bereaved person but also on the relationship to the deceased and circumstances of the loss as well as other social and cultural factors [43].

### 4.2. State of Research on Ecological Grief 

Empirical evidence on ecological grief is scarce. The authors of a systematic review of the mental health consequences of climate change-related events (e.g., heatwaves, floods; [2]) have identified several new terms to describe the emotional consequences of climate change, including ecological grief, but could not find any study that directly assessed how people react to changes to or losses of landscapes (e.g., deglaciation). However, they have confirmed the effects of extreme weather events on mental distress or disorders (e.g., sleep disturbances, PTSD, anxiety disorders). Cunsolo and Ellis [11] conducted as series of qualitative interviews with members of indigenous and farming communities in Canada and Australia to describe the phenomenology of ecological grief. A scoping review on solastalgia [19], a concept closely related to ecological grief (see 3.1), has revealed that the majority of research is based on qualitative interviews with groups particularly exposed to changes in their natural environment (e.g., farmers, coal mining community members). The few quantitative studies reported on the validation of the EDS, which contains a solastalgia subscale [21], or assessed symptoms of depression as a substitute for solastalgia, e.g., [44]. No study directly assessed the potential relation between solastalgia and mental health outcomes. Overall, there is mostly qualitative data on case descriptions of ecological grief or related constructs, the relation to mental health, mental health risks or environmental behavior remains unclear. 

## 5. Moderators and Mental Health Risks of Ecological Grief

### 5.1. Moderators 

There are several possible moderators of the development and actual experience of ecological grief [11,45]. People who retain close working, living or cultural relations with their natural environment are more likely to develop ecological grief. Groups such as farmers, mountaineers or indigenous peoples are more likely to be exposed directly to the weather and environmental effects of climate change (e.g., prolonged droughts, landscape changes after tornados). It has been proposed that certain groups are more vulnerable to the negative mental health effects of climate change, including those who have pre-existing disabilities, communities of color, older people, women, and children [3]. The extent to which these sociodemographic characteristics could increase the risk of ecological grief is currently unknown. Furthermore, the type of climate change experience (e.g., extreme weather events resulting in landscape changes vs. slow-moving deglaciation) may influence ecological grief. In bereavement research, the relationship to the deceased (particularly child and partner) and the type of death, mostly violent, unexpected, or multiple loss, have emerged as the strongest risk factors for PGD [38]. Finally, the personal and cultural value attributed to ecological loss is likely to modulate the experience of ecological grief. There might be differences between persons from more individualistic or collectivistic cultures, as shared cultural traditions may involve aspects of the natural environment and an ecological loss may thus include the loss of social connectedness. There are stark cross-cultural differences in the experience and expression of grief due to bereavement [46]. Moreover, people may form specific types of attachment to their living environment that influence the amount and duration of ecological grief.

### 5.2. Mental Health and Environmental Behavior Consequences

The consequences of ecological grief could be adaptive and/or maladaptive. As the experience of ecological grief can be painful and debilitating, it could pose a mental health risk [47] directly or by amplifying risk factors for mental disorders in several ways. First, the circumstances of particular ecological losses (e.g., forest loss after wildfires) could be perceived as traumatic. The experience of traumatic events has been identified as a risk factor for the development of various mental disorders such as depression or PTSD, particularly in the case of an accumulation of events [48,49]. It is likely that persons living in regions that are particularly vulnerable to climate change impacts will be exposed to multiple natural disasters over time. Thereby, it is possible that the presence of ecological grief may hinder persons from coping with the traumatic event and negatively influence meaning attribution. Second, withdrawal from social or recreational activities due to ecological grief but also the possible dislocation and splitting of communities after the loss of a particular place may lead to fewer social relationships and thus a reduction in social support over a prolonged period of time. The perception of low social support has emerged a major risk factor for the development and maintenance of mental disorders (e.g., PTSD; [50]). Third, ecological grief may be an additional source of stress that adds to current stressors such as economic hardships or somatic illness [3]. For example, it may exacerbate existing social problems, including food insecurity and overcrowded housing after the loss of a house in the context of an ecological loss (e.g., due to wildfires or tornados). Such compounded stress may increase the risk for mental health problems after ecological loss which has been well documented in the refugee field, whereby the negative mental health impact of stressful living conditions goes beyond the influence of cumulative traumatic experiences [51].

At the same time, ecological grief seems to be a normal and reasonable response to ecological loss. As emotional states motivate behavior tendencies [52], feelings associated with ecological grief (e.g., yearning, bitterness) could motivate environmental behavior. The ecological loss may give people a sense of hopelessness (e.g., ‘it doesn’t make a difference what I do’) and thus reduce the likelihood of active adaptation. However, research on psychological adaptation to climate change and its relation to environmental behavior has shown that some psychological distress in response to climate change-related environmental problems was positively associated with psychological coping or adaptation (e.g., expression of emotions in relation to environmental destruction), which in turn predicted engagement in pro-environmental behavior [53,54]. Predictions of concrete behavior depend on many factors [55] and it remains unclear whether climate change-related psychological distress is an indispensable precondition for behavior engagement or change [53,54]. Grief, anxiety and guilt are emotions that have been linked to man-made climate change, yet it remains a matter of further research whether they are linked to behavioral adaptions distinctively, and if so, to what extent.

## 6. Discussion

The loss of ecosystems, landscapes, species and ways of life becomes a lived experience for more and more people, whereby ecological grief is a natural response to such loss. There are unambiguous connections to bereavement research. Attachment features prominently in scientific explanations for bereavement and PGD [34,35,36] and homesickness [37] as it captures the resistance and disruption that occurs after separation by loss. A consequence of the separation is the necessity for adaptation to the changed situation. Therefore, it seems plausible that ecological grief is a phenomenon of yearning that encompasses typical grief-like psychological reactions to ecological loss, which may be similar in terms of determinants, manifestations and consequences to those associated with bereavement [39]. Over time, ecological grief may pose a risk factor for mental disorders or amplify risk factors for mental disorders (e.g., rumination about the circumstances of the loss, social withdrawal, additional sources of stress), but also motivate environment-related behavior. These conceptual linkages have not been discussed previously and not been addressed by any empirical research.

It has been proposed that ecological grief can be evoked by physical ecological loss, loss of environmental knowledge or anticipated future loss [11]. Research on the components and symptom profiles of PGD has reliably identified the disruption of personal identity (i.e., the feeling one has lost a part of one’s self or role confusion) as an integral part of the grief reaction, e.g., [41,56]. It seems plausible that feelings of yearning and longing after physical ecological loss are accompanied by a diminished personal identity in relation to the lost landscape, ecosystem, seasonal weather pattern, etc. On the other hand, anticipated future ecological loss refers in part to impending threats. Due to its orientation towards the future, anxiety is likely to be a predominant response to anticipated ecological loss. However, ecological grief may be triggered by future situations that relate to the current loss (e.g., a skier who lost snow cover may anticipate missing the landscape in the future on anticipated occasions such as vacations). The most universal and necessary trigger of ecological grief seems to be actual and past physical ecological loss. Overall, we propose that ecological grief is a phenomenon of yearning that refers to actual and past physical ecological loss or to future situations that trigger the actual/past loss, including all ecological losses such as home environments, ways of life, landscapes, species or ecosystems.

### Agenda for Future Research on Ecological Grief

There are several implications from this perspective discussion that warrant consideration in future research. Therefore, we propose several objectives for future research to move the study of ecological grief forward. First, it is essential to develop a measure of ecological grief. Such an instrument would facilitate consistency in understandings of the concept and allow for an empirical differentiation from the related construct of eco-anxiety but also more general climate change-related distress [54] and to empirically establish convergence with the solastalgia concept. Second, on a related note, it is important to compile a sort of taxonomy or list of types of ecological losses. This would also help to collect data to add conceptual clarity (e.g., is identity disruption a symptom of ecological grief or rather a type of ecological loss?). Third, it is pivotal that researchers systematically investigate the possible determinants (e.g., degree of place attachment, type of climate change experience), mental health correlates and behavioral consequences of actual and past ecological loss in different populations and sociodemographic groups (e.g., representative samples of general populations, displaced persons, indigenous peoples, youth, climate strike participants) by employing quantitative besides qualitative methods [19]. Finally, it is strongly advised that researchers develop and evaluate strategies to reduce the mental health risks possibly associated with ecological grief. Most people suffering from ecological grief will function well and adapt to environmental changes, however, some people may need psychosocial support, particularly with regard to possible social problems and debilitating rumination. Several solution-focused coping strategies could be helpful. For example, joining groups for climate change-related mental well-being [45] or climate change awareness groups may help to express feelings of sadness, anxiety or guilt and at the same time enhance social support. The cognitive restructuring of excessive feelings of guilt/responsibility for the loss could be fostered to regain a sense of control. Such groups may be further enriched with psychoeducational information about grieving norms in different cultures and emotion recognition and regulation but also with practicing self-management skills to reduce debilitating symptoms of ecological grief and strategies to foster adaptation to the loss [57,58] and general well-being, e.g., Gander, F., et al. [59].

## 7. Conclusions

The experience of ecological grief is likely to become more frequent around the world. This has already been recognized in popular culture. However, there are almost no empirical studies on ecological grief and its possible impacts on mental health and mental health risks. The contextualization of ecological grief within the field of bereavement research and its conceptual differentiation from related concepts of climate change-induced distress could help to systematically follow an agenda for future research that is embedded in bereavement research. This will deepen our understanding of climate change-related psychological phenomena beyond the well-known mental health consequences of disasters and contribute to decreasing mental suffering in people affected by ecological grief, while increasing their potential for restorative action.

## Data Availability

Not applicable.
